# A 1:2 co-crystal of 2,2′-thiodi­benzoic acid and tri­phenyl­phosphane oxide: crystal structure, Hirshfeld surface analysis and computational study

**DOI:** 10.1107/S205698901801544X

**Published:** 2018-11-09

**Authors:** Sang Loon Tan, Edward R. T. Tiekink

**Affiliations:** a Research Centre for Crystalline Materials, School of Science and Technology, Sunway University, 47500 Bandar Sunway, Selangor Darul Ehsan, Malaysia

**Keywords:** crystal structure, thio­ether, phosphane oxide, hydrogen bonding, Hirshfeld surface analysis

## Abstract

The asymmetric unit of the title co-crystal, 2,2′-thiodi­benzoic acid–tri­phenyl­phosphane oxide (1/2), C_14_H_10_O_4_S·2C_18_H_15_OP, comprises two mol­ecules of 2,2′-thiodi­benzoic acid [TDBA; systematic name: 2-[(2-carb­oxy­phen­yl)sulfan­yl]benzoic acid] and four mol­ecules of tri­phenyl­phosphane oxide [TPPO; systematic name: (di­phenyl­phosphor­yl)benzene]. The two TDBA mol­ecules are twisted about their di­sulfide bonds and exhibit dihedral angles of 74.40 (5) and 72.58 (5)° between the planes through the two SC_6_H_4_ residues. The carb­oxy­lic acid groups are tilted out of the planes of the rings to which they are attached forming a range of CO_2_/C_6_ dihedral angles of 19.87 (6)–60.43 (8)°. Minor conformational changes are exhibited in the TPPO mol­ecules with the range of dihedral angles between phenyl rings being −2.1 (1) to −62.8 (1)°. In the mol­ecular packing, each TDBA acid mol­ecule bridges two TPPO mol­ecules *via* hy­droxy-O—H⋯O(oxide) hydrogen bonds to form two three-mol­ecule aggregates. These are connected into a three-dimensional architecture by TPPO-C—H⋯O(oxide, carbon­yl) and TDBA-C—H⋯(oxide, carbon­yl) inter­actions. The importance of H⋯H, O⋯H/H⋯O and C⋯H/H⋯C contacts to the calculated Hirshfeld surfaces has been demonstrated. In terms of individual mol­ecules, O⋯H/H⋯O contacts are more important for the TDBA (*ca* 28%) than for the TPPO mol­ecules (*ca* 13%), as expected from the chemical composition of these species. Computational chemistry indicates the four independent hy­droxy-O—H⋯O(oxide) hydrogen bonds in the crystal impart about the same energy (*ca* 52 kJ mol^−1^), with DTBA-phenyl-C—H⋯O(oxide) inter­actions being next most stabilizing (*ca* 40 kJ mol^−1^).

## Abstract 2

The asymmetric unit of the title co-crystal comprises two twisted mol­ecules of 2,2′-thiodi­benzoic acid and four mol­ecules of tri­phenyl­phosphane oxide. The three-dimensional mol­ecular packing is stabilized by hy­droxy-O—H⋯O(oxide) hydrogen bonds and TPPO-C—H⋯O(oxide, carbon­yl) and TDBA-C—H⋯(oxide, carbon­yl) inter­actions.

## Chemical context   

2-Thio­salicylic acid, also known as 2-mercapto­benzoic acid, being an analogue to salicylic acid, has many applications. In medicine, is dianion is found in the salt Na[EtHg(SC_6_H_4_CO_2_)], which displays anti-fungal and anti-septic activities (Bigham & Copes, 2005 ▸). Other uses include as anti-corrosion agents (Chien *et al.*, 2012 ▸), as reactive agents or modifiers for nanoparticles and electrochemical sensing (Cang *et al.*, 2017 ▸; Sikarwar *et al.*, 2014 ▸), as catalysts for organic syntheses (Yang *et al.*, 2018 ▸; Selig & Miller, 2011 ▸) as well as being the precursor for some anti-viral and anti-microbial agents (Saha *et al.*, 2017 ▸). The compound readily coordinates a wide variety of metals, in both neutral and anionic form, due to the presence of both hard (oxygen) and soft (sulfur) donor atoms and exhibits different modes of coordination. Very recent reviews of the coordination chemistry of 2-thio­salicylic acid (Wehr-Candler & Henderson, 2016 ▸) and the isomeric 3- and 4-species (Tiekink & Henderson, 2017 ▸) are available. However, a restriction in the chemistry of this mol­ecule is found as it can undergo various pH-dependent transformations, *i.e*. it remains intact in acidic condition but may be oxidized to form 2,2′-di­thiodi­benzoic acid at neutral pH. For example and relevant to the present contribution, are studies of co-crystal formation between 2-thio­salicylic acid and bipyridyl-type mol­ecules (Broker & Tiekink, 2007 ▸) whereby 2-thio­salicylic acid was oxidized to 2,2′-di­thiodi­benzoic acid during co-crystallization. During attempts to react 2-thio­salicylic acid with copper(I) chloride in the presence of two equivalents of tri­phenyl­phosphane, motivated by the desire to prepare analogues of phosphanecopper(I) di­thio­carbamate derivatives which exhibit promising anti-bacterial activity (Jamaludin *et al.*, 2016 ▸), the title co-crystal was isolated, *i.e*. the 1:2 co-crystal of 2,2′-thiodi­benzoic acid and tri­phenyl­phosphane oxide (I). Unexpectedly, both organic reagents were found to have oxidized in the presence of copper(I) chloride in aceto­nitrile solution under neutral conditions. While the actual mechanism remains unclear, a very recent study describes related synthetic outcomes (Gorobet *et al.*, 2018 ▸). Herein, the crystal and mol­ecular structures, the analysis of the calculated Hirshfeld surface and calculation of the inter­action energies through a computational approach for (I) are described.
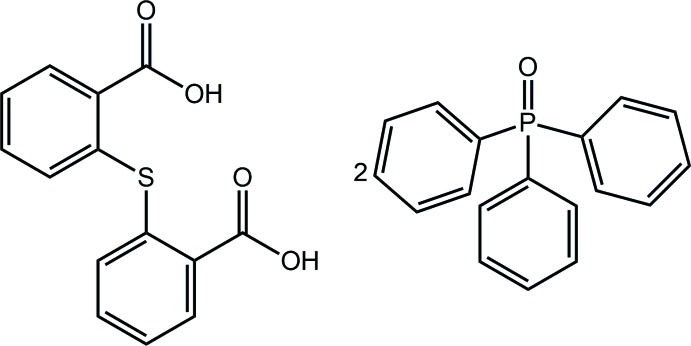


## Structural commentary   

**Figure 1:**
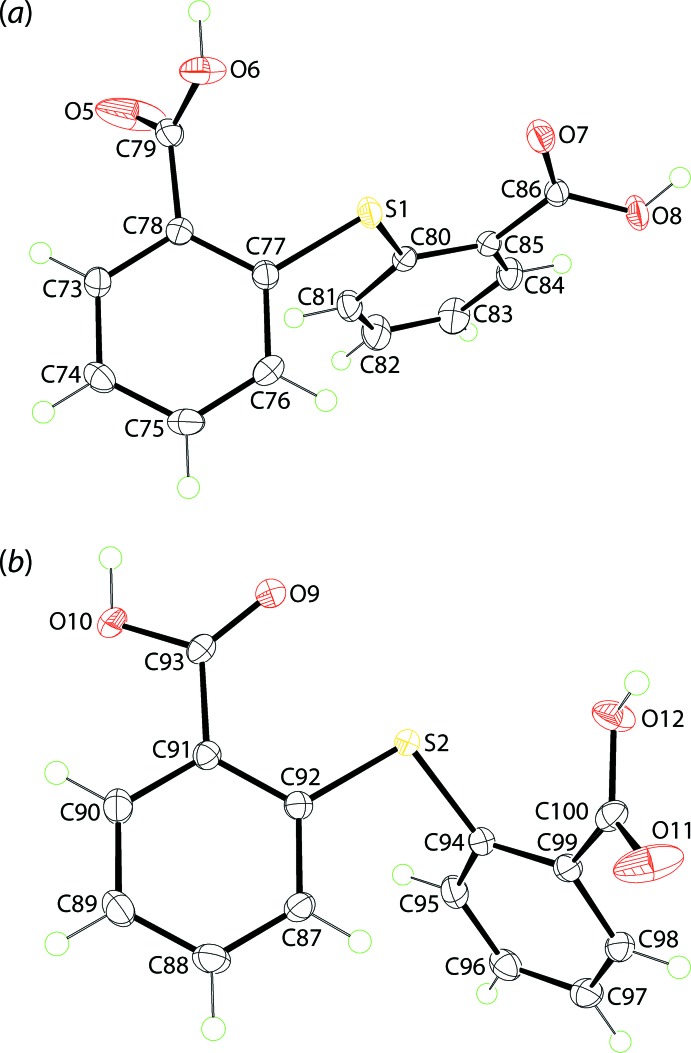
The mol­ecular structures of the two independent mol­ecules of 2,2′-thiodi­benzoic acid in the asymmetric unit of (I), showing the atom-labelling scheme and displacement ellipsoids at the 70% probability level.

**Figure 2:**
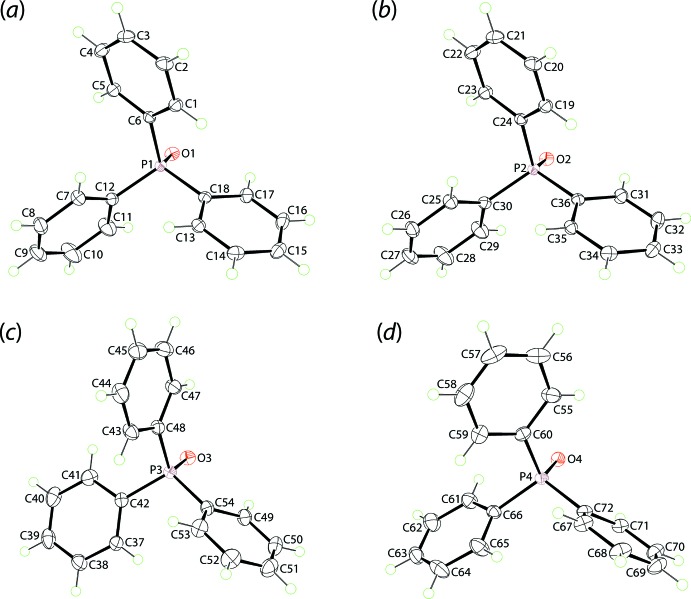
The mol­ecular structures of the four independent mol­ecules of tri­phenyl­phosphane oxide in the asymmetric unit of (I), showing the atom-labelling scheme and displacement ellipsoids at the 70% probability level.

X-ray crystallography reveals the title co-crystal to comprise 2,2′-thiodi­benzoic acid (TDBA) and tri­phenyl­phosphane oxide (TPPO) in the ratio 1:2, but with two independent TDBA mol­ecules, Fig. 1 ▸, and four independent TPPO mol­ecules, Fig. 2 ▸, in the asymmetric unit.

**Figure 3:**
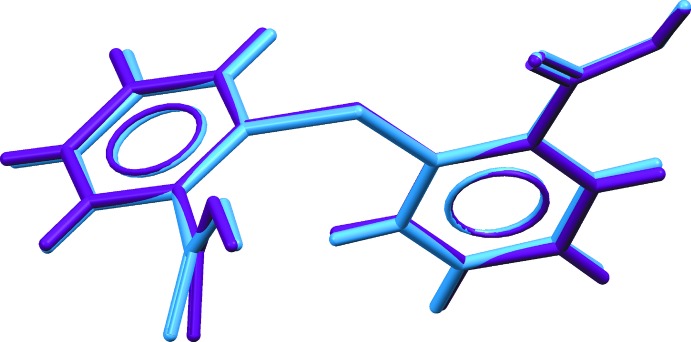
An overlay diagram of the two independent mol­ecules of 2,2′-thiodi­benzoic, with S1-mol­ecule (purple) and S2-mol­ecule (light-blue) superimposed so that a pair thio­phenyl moieties are coincident.

Each TDBA mol­ecule comprises two benzoic acid residues connected in the 2-positions by a sulfur bridge. The confirmation of the presence of carb­oxy­lic acid groups is readily seen in the disparity in the C—O(hy­droxy) and C=O(carbon­yl) bond lengths with the minimum difference seen for the C100=O11 and C100—O12 bonds of 1.3126 (15) and 1.2075 (16) Å, respectively. As expected, the thio­phenyl residues are almost planar with the maximum r.m.s. deviation of 0.053 Å being found for the S1,C80–C85 atoms. The thio­phenyl rings are deviated from the perfect perpendicular bis­ector with dihedral angles of 74.40 (5) and 72.58 (5)° for the S1- and S2-mol­ecules, respectively. Finally, the O6-, O8-, O10- and O12- carb­oxy­lic acid groups are tilted from the phenyl rings they are connected to by 60.43 (8), 24.24 (7), 19.87 (6) and 45.78 (7)°, respectively. That there are no major conformational differences between the mol­ecules is evidenced from the overlay diagram of Fig. 3 ▸ (r.m.s. deviation = 0.118 Å).

The mol­ecular structures of the TPPO coformers are more rigid. This is seen in the O—P—C—C torsion angles, which range from 17.7 (1) to 61.6 (1), 19.8 (1) to 61.5 (1), −2.1 (1) to −62.8 (1) and −19.2 (1) to −44.5 (1)° for the P1–P4-mol­ecules, respectively. In the same way, the P=O bond lengths span an experimentally equivalent range, *i.e*. 1.4975 (8) [P4=O4] to 1.5018 (8) Å [P1=O1].

## Supra­molecular features   

**Figure 4:**
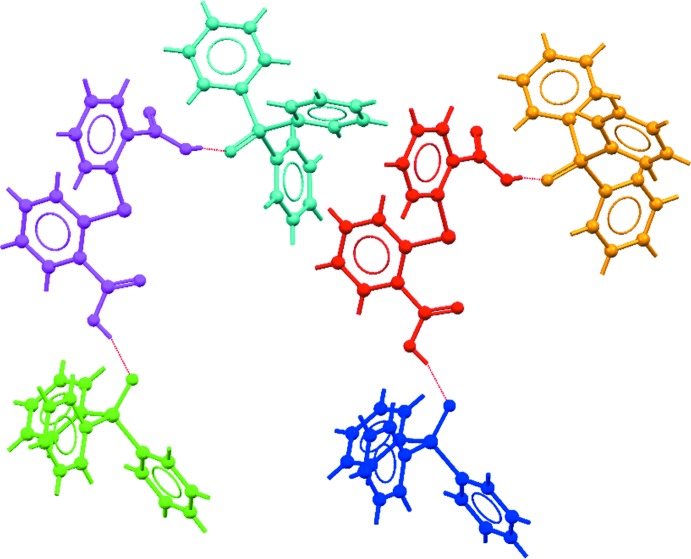
The two three-mol­ecule aggregates in the crystal of (I). The hy­droxy-O—H⋯O(oxide) hydrogen bonds are shown as red dashed lines. Colour code: S1-containing mol­ecule, purple; S2, red; P1, green; P2, blue; P3, yellow; P4, light-blue.

**Figure 5:**
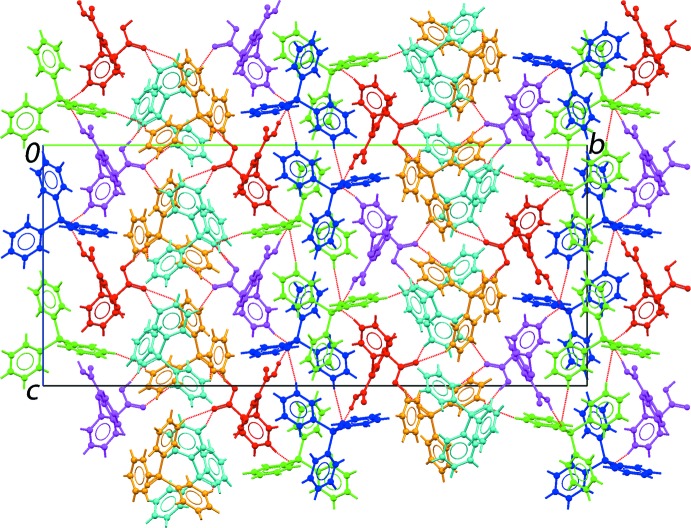
A view of the unit-cell contents shown in projection down the *a* axis. The mol­ecules are colour-coded as for Fig. 4 ▸.

Geometric parameters characterizing the identified (*PLATON*; Spek, 2009 ▸) inter­atomic contacts in the crystal of (I) are given in Table 1 ▸. The most prominent feature of the mol­ecular packing is the formation of hy­droxy-O—H⋯O(oxide) hydrogen bonds. These occur so that each mol­ecule of 2,2′-thiodi­benzoic acid (TDBA) links two tri­phenyl­phosphane oxide (TPPO) mol­ecules to form a pair of three-mol­ecule aggregates with a 13-membered, linear {O⋯HOC_3_SC_3_OH⋯O} heterosynthon as illustrated in Fig. 4 ▸. These aggregates are connected into a three-dimensional architecture by a large number of C—H⋯O inter­actions. Two of these contacts, *i.e*. TPPO-C47—H⋯O11(carbon­yl) and TPPO-C71—H⋯O5(carbon­yl), operate in concert with hy­droxy-O12—H⋯O3(oxide) and hy­droxy-O6—H⋯O4(oxide) hydrogen bonds, respectively, to close a nine-membered {HC_2_PO⋯HOCO⋯} synthon. The C—H⋯O contacts are of the type TPPO-C—H⋯O(oxide, carbon­yl) and TDBA-C—H⋯O(oxide, carbon­yl), Table 1 ▸. In addition to participating in hy­droxy-O—H⋯O(oxide) hydrogen bonds, each of the O1–O3 atoms of TPPO form an additional C—H⋯O(oxide) contact whereas the O4 atom participates in two such inter­actions. One carbonyl group of each TDBA mol­ecule, *i.e*. the O5 and O11 atoms, participates in two C—H⋯O(carbon­yl) inter­actions, leaving no formal role for the carbonyl-O7 and O9 atoms in the mol­ecular packing. A view of the unit-cell contents is shown in Fig. 5 ▸.

In terms of distinguishing between mol­ecules based on inter­molecular contacts, the carbonyl-O5 atom of DTBA accepts C—H⋯O inter­actions from phenyl rings derived from TPPO and DTBA, whereas the carbonyl-O11 atom accepts contacts from TPPO only. The DPPO-O4 atom is distinct from the O1–O3 atoms based on the number of inter­actions it forms. In common with the O4 atom, O3 accepts a C—H⋯O inter­action from TPPO, whereas each of the O1 and O2 participates in DTBA-C—H⋯O contacts.

## Hirshfeld surface analysis   

**Figure 6:**
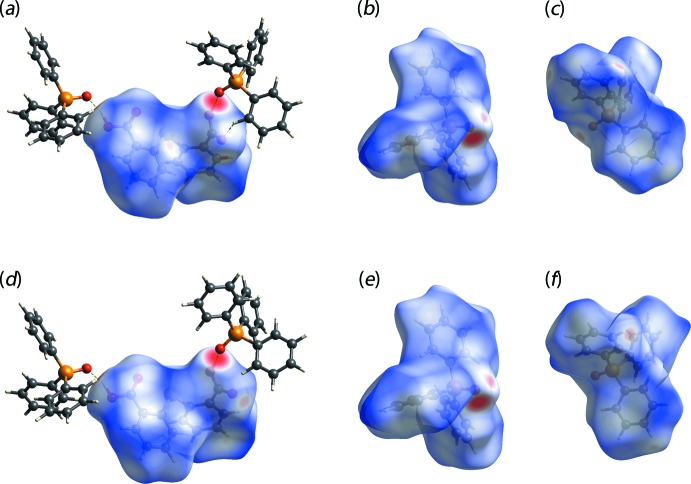
Views of the Hirshfeld surfaces mapped over *d*_norm_ for components of (I) for the: (*a*) S1-DTBA mol­ecule hydrogen bonded (red dashed lines) to the P1- (left) and P4-TPPO mol­ecules, (*b*) P1-TPPO, (*c*) P4-TPPO, (*d*) S2-DTBA mol­ecule hydrogen bonded to the P2- (left) and P3-TPPO mol­ecules, (*e*) P2-TPPO and (*f*) P3-TPPO. The surfaces in (*a*)–(*c*) are mapped over the range −0.766 to 1.446 a.u., and those in (*d*)–(*f*) over the range −0.766 to 1.563 a.u.

The independent 2,2′-thiodi­benzoic acid (TDBA) and tri­phenyl­phosphane oxide (TPPO) mol­ecules of (I) were subjected to Hirshfeld surface analysis following a literature precedent on a multi-component crystal (Jotani *et al.*, 2018 ▸) to further understand the nature of the inter­molecular inter­actions in the crystal. As shown in Fig. 6 ▸(*a*)–(*f*), the pair of TDBA-S1 and -S2 mol­ecules, shown with the respective pairs of hydrogen bonded TPPO mol­ecules, as well as the TPPO-P1–P4 mol­ecules exhibit some similarities especially on the prominent close contacts as represented by the intense red regions on the corresponding *d*_norm_ surface mappings, which are mainly dominated by hydroxy-O—H⋯O(oxide) inter­actions.

**Figure 7:**
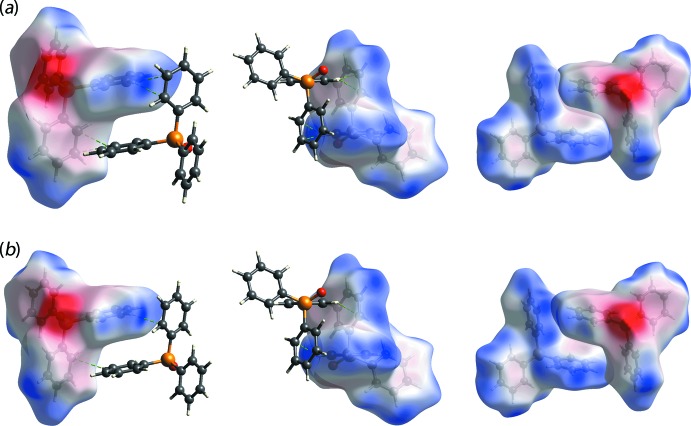
Different views of the Hirshfeld surfaces mapped over electrostatic potential for the centrosymmetrically related mol­ecules of TPPO inter­acting *via* semi-localized phenyl-C—H⋯π(phen­yl) inter­actions: (*a*) P1-TPPO, in the range of −0.100 to 0.041 a.u. and (*b*) P2-TPPO mol­ecules (−0.100 to 0.041 a.u.).

Upon close inspection on the surface mapping, minor differences are observed between the pair of TDBA mol­ecules. Specifically, a diminutive red spot is observed near one of the terminal carb­oxy­lic groups of the S1-mol­ecule arising from a TPPO-phenyl-C—H⋯O(carbonyl) inter­action but, no such contact is apparent for the S2-mol­ecule. As for the two pairs of TPPO mol­ecules, the significant difference between the TPPO-P1 and -P4 mol­ecules, linked to S1-DTBA, and the TPPO-P2 and P3 mol­ecules, linked to the S2-TDBA, is the presence of additional red spots on the surface mapping of the phenyl rings for P1- and P2-mol­ecules in contrast to their P3- and P4-containing counterparts. This difference may be attributed to the complementary phenyl-C—H⋯π(phen­yl) inter­actions between centrosymmetrically-related mol­ecules, as illustrated in Fig. 7 ▸ and tabulated in Table 2 ▸. Here, the inter­acting H10 and H28 atoms are directed towards two carbon atoms of a symmetry-related ring so that the inter­actions are best described as being semi-localized as opposed to delocalized, which corresponds to the situation where the inter­acting hydrogen atom is equally separated from all six carbon atoms of the ring (Schollmeyer *et al.*, 2008 ▸).

**Figure 8:**
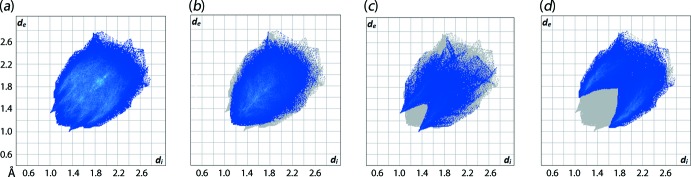
(*a*) The full two-dimensional fingerprint plot for (I) and (*b*)–(*d*) those delineated into H⋯H, O⋯H/H⋯O and C⋯H/H⋯C contacts, respectively.

**Figure 9:**
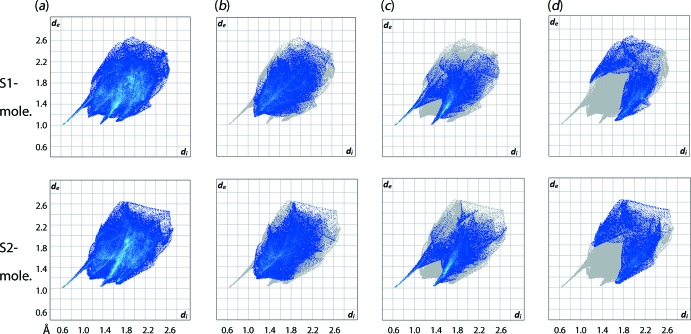
(*a*) The full two-dimensional fingerprint plot for the two independent TDBA mol­ecules in (I) and (*b*)–(*d*) those delineated into H⋯H, O⋯H/H⋯O and C⋯H/H⋯C contacts, respectively.

**Figure 10:**
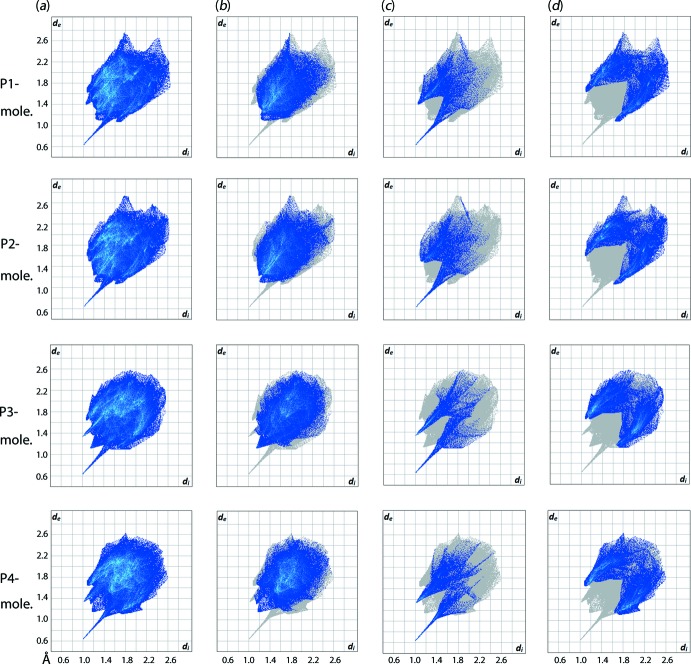
(*a*) The full two-dimensional fingerprint plot for the four independent TPPO mol­ecules in (I) and (*b*)–(*d*) those delineated into H⋯H, O⋯H/H⋯O and C⋯H/H⋯C contacts, respectively.

Qu­anti­tative evaluation of the Hirshfeld surfaces by the combination of the *d*_i_ and *d*_e_ (*i* is inter­nal and *e* is external to the surface) contact distances in inter­vals of 0.01 Å gives the overall two-dimensional fingerprint plots for the entire asymmetric unit of (I), Fig. 8 ▸(*a*), and each of the individual TDBA, Fig. 9 ▸(*a*), and TPPO, Fig. 10 ▸(*a*), mol­ecules. Further, these can be delineated into specific contacts (McKinnon *et al.*, 2007 ▸) and Figs. 9 ▸–10 ▸(*b*)–(*d*) give fingerprint plots delineated into H⋯H, O⋯H/H⋯O and C⋯H/H⋯C contacts. The relative contributions of these contacts to the surfaces is given in Table 3 ▸.

The overall fingerprint plot for (I), Fig. 8 ▸ *a*, is quite different for the individual components, Figs. 9 ▸–10 ▸ *a*, as the former is a sum of all the individual surface contacts, which differ for the individual mol­ecules. As expected, the same is true for the corresponding decomposed fingerprint plots. The major contribution to the overall surface of (I), *i.e*. 49.4%, comes from H⋯H contacts. The O⋯H/H⋯O contacts (*d*_e_ + *d*_i_ ∼ 2.34 Å) make a significant contribution at 13.7%, while the C⋯H/H⋯C inter­actions (*d*_e_ + *d*_i_ ∼ 2.66 Å), at 30.1%, play a more prominent role.

The formation of the 13-membered {O⋯HOC_3_SC_3_OH⋯O} heterosynthon, Fig. 4 ▸, is clearly reflected in the corresponding full fingerprint plots of the individual mol­ecules Figs. 9 ▸–10 ▸(*a*), which exhibit an almost identical claw-like fingerprint profile but arranged in the exact reverse order, *i.e*. Fig. 9 ▸(*a*) *cf*. Fig. 10 ▸(*a*). Among all the close inter­actions, H⋯H contacts, Figs. 8 ▸–9 ▸ *b*, represent the dominant inter­actions to the individual surfaces, *i.e*. 41–42% for the TDBA mol­ecules and 49–51% for the DPPO mol­ecules, and exhibit *d*_e_ + *d*_i_ contact distances ranging from 2.24 to 2.38 Å which is very close to the sum of van der Waals radii of 2.4 Å.

The O⋯H hydrogen bonds constitute the strongest among all inter­actions present in the co-crystal and lead to formation of asymmetric, forceps-like profiles in the corresponding decomposed fingerprint plots, Figs. 9 ▸–10 ▸(*c*). These feature two tips – one at relatively short *d*_e_ + *d*_i_ ∼1.6 Å that can be attributed to the hydroxy-H⋯O(oxide) hydrogen bonds for the S1- and S2-TDBA mol­ecules, Fig. 10 ▸(*c*), or oxide-O⋯H(hydrox­y) hydrogen bonds for P1–P4-TPPO. The other tip has a relatively long *d*_e_ + *d*_i_ value of ∼2.4 Å and arises as a result of hy­droxy-O⋯H(phen­yl) contacts for S1- and S2-TDBA or phenyl-H⋯O(hydrox­y) for P1–P4-TPPO. The O⋯H/H⋯O contacts constitute the second most dominant inter­actions for the TDBA mol­ecules and third most for the TPPO mol­ecules, Table 3 ▸.

Similar to the H⋯H contacts, the C⋯H/H⋯C inter­actions contribute weakly to the mol­ecular packing of the co-crystal as evidenced from the *d*_e_ + *d*_i_ distance range of 2.7–2.8 Å, *i.e*. close to the sum of van der Waals radii of 2.9 Å, despite the contacts constituting the third most dominant inter­action in the TDBA mol­ecules (*ca* 22%) and being the second most dominant for the TPPO mol­ecules (*ca* 32%). An exception to the trend is found for the P1- and P2-TPPO mol­ecules, which display relatively short contact distances at *ca* 2.6 Å owing to the formation of C—H⋯π inter­actions as discussed above.

In summary the Hirshfeld surface analysis on (I), with six individual constituents, was able to distinguish between these in terms of different inter­molecular inter­actions, akin to the recently reported analysis of a structure with four independent cation/anion pairs (Jotani *et al.*, 2018 ▸).

## Computational study   

The co-crystal was subjected to inter­molecular inter­action energy calculations using CE-B3LYP/6-31G(*d*,*p*) available in *Crystal Explorer* (version 17; Turner *et al.*, 2017 ▸), with the crystal geometry being used as the input but, with hydrogen-atom positions normalized to the standard neutron diffraction values. By default, a cluster of mol­ecules (defined as density matrices) would need to be generated by applying crystallographic symmetry operations with respect to a selected central mol­ecule (density matrix) within the radius of 3.8 Å for inter­action energy calculation (Turner *et al.*, 2014 ▸). However, as the co-crystal contains multiple independent mol­ecules in the asymmetric unit, a cluster of mol­ecules was first generated surrounding the S1-mol­ecule of TDBA for the calculation and then the procedure was repeated for the cluster of mol­ecules surrounding the S2-mol­ecule. The total inter­molecular energy is the sum of energies of four main components comprising electrostatic, polarization, dispersion and exchange-repulsion with a scale factors of 1.057, 0.740, 0.871 and 0.618, respectively (Mackenzie *et al.*, 2017 ▸).

**Figure 11:**
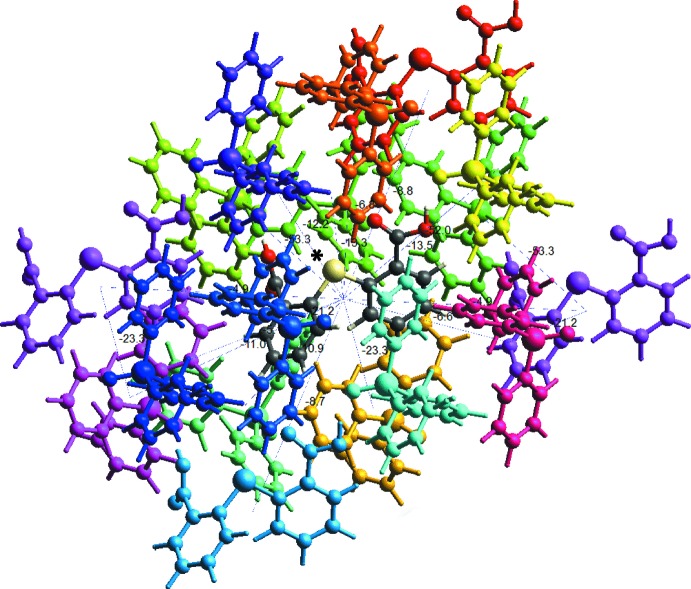
The inter­action energy framework about the S1-mol­ecule of DTBA (indicated by an asterisk) viewed along the *b*-axis direction.

Selected results obtained from the inter­action energy calculations involving the DTBA mol­ecules as reference mol­ecules are tabulated in Table 4 ▸ and the environment about the S1-mol­ecule of TDBA is shown in Fig. 11 ▸. As expected, O—H⋯O hydrogen bonding inter­actions give the greatest energies among the close contacts present in the crystal. The total inter­molecular energy (*E*_tot_) of the hy­droxy-O—H⋯O(oxide) hydrogen bonds is consistent across the series and lies in the range −50.7 to −53.3 kJ mol^−1^. The other close contacts which exerts a relatively strong influence in the energy frameworks of the co-crystal are DTBA-phenyl-C—H⋯O(oxide) inter­actions, with the *E*_tot_ amounting of *ca* −40 kJ mol^−1^, Table 4 ▸.

## Database survey   

The only other structure of 2,2′-thiodi­benzoic acid in the literature is that of the pure compound (Dai *et al.*, 2005 ▸). While this presents essentially the same features as for the two independent mol­ecules in (I), the dihedral angle between the thio­phenyl rings is up to 4° smaller at 68.0 (2)°, and the tilts of the carb­oxy­lic acid groups are less pronounced at 6.9 (5) and 29.8 (5)°.

**Figure 12:**
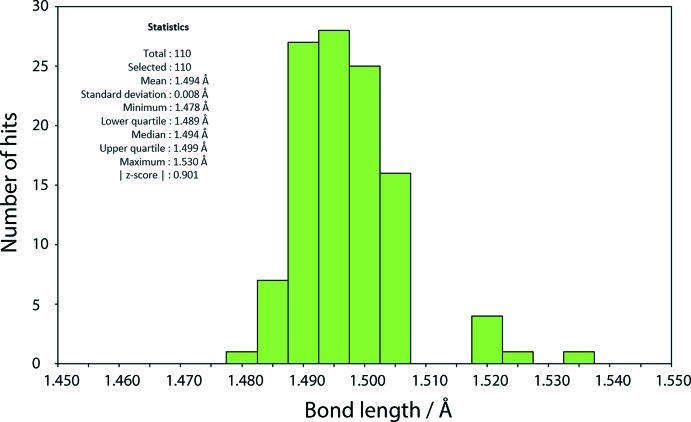
Statistical data on the P=O bond lengths as calculated by *Mogul* (Bruno *et al.*, 2004 ▸)

A survey of the Cambridge Structural Database (Groom *et al.*, 2016 ▸), revealed 110 mol­ecules of (non-coordinated) tri­phenyl­phosphane oxide. A plot of the retrieved P=O bond lengths is shown in Fig. 12 ▸. The mean value found for the P=O bond length is 1.494 Å with a standard deviation of 0.008 Å, with the minimum and maximum bond lengths being 1.478 (3) and 1.530 (7) Å, found in the multi-component structures of NUCHIC (Okawa *et al.*, 1997 ▸) and DUYXUQ (Arens *et al.*, 1986 ▸), respectively. In the latter structure, charge-assisted hydrogen bonds are formed between Ph_3_P=O and Ph_3_P=O^(+)^H. The observed P=O bond lengths in (I), *i.e*. in the range 1.4975 (8) to 1.5018 (8) Å are at the lower end of the range of such bonds.

## Synthesis and crystallization   

All chemical precursors were of reagent grade and used as received without purification. Thio­salicylic acid (Merck; 0.154 g, 0.001 mol) and tri­phenyl­phosphane (Merck; 0.262 g, 0.002 mol) were dissolved in aceto­nitrile (40 ml) and the mixture subsequently added into an aceto­nitrile solution (25 ml) of copper(I) iodide (Merck; 0.19 g, 0.001 mol). The reaction mixture was stirred for 1 h at room temperature before the white product was filtered, washed with cold ethanol and dried *in vacuo*. The filtrate was left at room temperature, yielding colourless prisms after 1 week; Yield 74%. M.p. 457.7–459.2 K. IR (cm^−1^): 3062 ν(C—H), 1693 ν(COO), 1236 ν(P=O), 1116 ν(P—Ar), 719 δ(P—C), 617 ν(C—S).

## Refinement   

Crystal data, data collection and structure refinement details are summarized in Table 5 ▸. The carbon-bound H atoms were placed in calculated positions (C—H = 0.93 Å) and were included in the refinement in the riding-model approximation, with *U*_iso_(H) set to 1.2*U*_eq_(C). The oxygen-bound H atoms were located from difference Fourier maps and refined without constraint. Owing to poor agreement, three reflections, *i.e*. (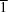 5 9), (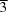 15 3) and (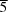 7 9), were omitted from the final cycles of refinement.

## Supplementary Material

Crystal structure: contains datablock(s) global, I. DOI: 10.1107/S205698901801544X/hb7782sup1.cif

Structure factors: contains datablock(s) I. DOI: 10.1107/S205698901801544X/hb7782Isup2.hkl

Supporting information file. DOI: 10.1107/S205698901801544X/hb7782Isup3.cml

CCDC reference: 1876525

Additional supporting information:  crystallographic information; 3D view; checkCIF report

## Figures and Tables

**Table 1:** Hydrogen-bond geometry (Å, °)

*D*—H⋯*A*	*D*—H	H⋯*A*	*D*⋯*A*	*D*—H⋯*A*
O6—H6*O*⋯O4	0.95 (2)	1.66 (2)	2.6070 (12)	171 (2)
O8—H8*O*⋯O1^i^	0.90 (2)	1.70 (2)	2.5763 (12)	163 (2)
O10—H10*O*⋯O2^ii^	0.91 (2)	1.72 (2)	2.6077 (12)	163 (2)
O12—H12*O*⋯O3^iii^	0.90 (2)	1.71 (2)	2.5978 (12)	170.9 (19)
C16—H16⋯O4	0.93	2.53	3.3333 (15)	144
C44—H44⋯O4^iv^	0.93	2.43	3.2404 (17)	145
C52—H52⋯O11^v^	0.93	2.49	3.3231 (16)	149
C62—H62⋯O11^i^	0.93	2.51	3.367 (2)	153
C64—H64⋯O5^vi^	0.93	2.46	3.263 (2)	144
C68—H68⋯O3^vi^	0.93	2.55	3.2747 (17)	135
C71—H71⋯O5	0.93	2.59	3.2765 (18)	131
C75—H75⋯O2^i^	0.93	2.41	3.1184 (16)	133
C96—H96⋯O1	0.93	2.49	3.1832 (15)	132

Symmetry codes: (i) 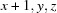; (ii) 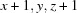; (iii) 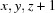; (iv) 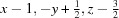; (v) 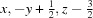; (vi) 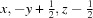.

**Table 2:** Summary of short C⋯H inter­atomic contacts (Å) in (I)

Contact	Separation	Symmetry operation
C13⋯H10	2.80	1 − *x*, 1 − *y*, 1 − *z*
C14⋯H10	2.84	1 − *x*, 1 − *y*, 1 − *z*
C35⋯H28	2.77	-*x*, 1 − *y*, −*z*
C34⋯H28	2.94	-*x*, 1 − *y*, −*z*

**Table 3:** Percentage contributions of inter­atomic contacts to the Hirshfeld surface for (I) and for the the individual TDBA and DPPO mol­ecules

Contact	Percentage contribution						
	overall	S1-TDBA	S2-TDBA	P1-DPPO	P2-DPPO	P3-DPPO	P4-DPPO
H⋯H	49.4	42.3	40.7	49.8	49.6	49.7	51.4
O⋯H/H⋯O	13.7	28.1	28.1	14.1	13.6	11.7	12.7
C⋯H/H⋯C	30.1	21.9	23.4	30.2	30.9	33.4	31.3

**Table 4:** Inter­action energies (kJ mol^−1^) for selected close contacts

contact	*E*_electrostatic_	*E*_polarization_	*E*_dispersion_	*E*_exchange-repulsion_	*E*_total_	Symmetry operation
O6—H6*O*⋯O4	−76.5	−19.4	−17.8	95.2	−52.0	*x*, *y*, *z*
O8—H8*O*⋯O1	−72.8	−19.2	−14.9	82.3	−53.3	1 + *x*, *y*, *z*
O10—H10*O*⋯O2	−70.5	−18.3	−16.4	83.8	−50.7	1 + *x*, *y*, 1 + *z*
O12—H12*O*⋯O3	−72.3	−19.2	−13.9	81.2	−52.5	*x*, *y*, 1 + *z*
C75—H75⋯O2	−16.8	−6.6	−41.6	29.8	−40.4	1 + *x*, *y*, *z*
C96—H96⋯O1	−15.2	−6.1	−42.0	27.9	−40.0	*x*, *y*, *z*

**Table 5:** Experimental details

Crystal data	
Chemical formula	4C_18_H_15_OP·2C_14_H_10_O_4_S
*M*_r_	1661.64
Crystal system, space group	Monoclinic, *P*2_1_/*c*
Temperature (K)	100
*a*, *b*, *c* (Å)	10.7085 (1), 41.9751 (2), 18.9268 (1)
β (°)	101.490 (1)
*V* (Å^3^)	8336.92 (10)
*Z*	4
Radiation type	Cu *K*α
μ (mm^−1^)	1.83
Crystal size (mm)	0.17 × 0.16 × 0.09

Data collection	
Diffractometer	XtaLAB Synergy, Dualflex, AtlasS2
Absorption correction	Gaussian (*CrysAlis PRO*; Rigaku OD, 2018 ▸)
*T*_min_, *T*_max_	0.653, 1.000
No. of measured, independent and observed [*I* > 2σ(*I*)] reflections	94929, 17036, 15740
*R*_int_	0.025
(sin θ/λ)_max_ (Å^−1^)	0.630

Refinement	
*R*[*F* ^2^ > 2σ(*F* ^2^)], *wR*(*F* ^2^), *S*	0.033, 0.092, 1.04
No. of reflections	17036
No. of parameters	1079
H-atom treatment	H atoms treated by a mixture of independent and constrained refinement
Δρ_max_, Δρ_min_ (e Å^−3^)	0.42, −0.55

Computer programs: *CrysAlis PRO* (Rigaku OD, 2018 ▸), *SHELXT* (Sheldrick, 2015*a* ▸), *SHELXL* (Sheldrick, 2015*b* ▸), *ORTEP-3 for Windows* (Farrugia, 2012 ▸), *OLEX2* (Dolomanov *et al.*, 2009 ▸), *Mercury* (Macrae *et al.*, 2006 ▸) and *publCIF* (Westrip, 2010 ▸).

## supplementary crystallographic information

### Crystal data

**Table:**

4C_18_H_15_OP·2C_14_H_10_O_4_S	*F*(000) = 3472
*M**_r_* = 1661.64	*D*_x_ = 1.324 Mg m^−^^3^
Monoclinic, *P*2_1_/*c*	Cu *K*α radiation, λ = 1.54184 Å
*a* = 10.7085 (1) Å	Cell parameters from 52739 reflections
*b* = 41.9751 (2) Å	θ = 4.2–75.9°
*c* = 18.9268 (1) Å	µ = 1.83 mm^−^^1^
β = 101.490 (1)°	*T* = 100 K
*V* = 8336.92 (10) Å^3^	Prism, colourless
*Z* = 4	0.17 × 0.16 × 0.09 mm

### Data collection

**Table:**

XtaLAB Synergy, Dualflex, AtlasS2 diffractometer	17036 independent reflections
Radiation source: micro-focus sealed X-ray tube, PhotonJet (Cu) X-ray Source	15740 reflections with *I* > 2σ(*I*)
Mirror monochromator	*R*_int_ = 0.025
Detector resolution: 5.2558 pixels mm^-1^	θ_max_ = 76.2°, θ_min_ = 3.2°
ω scans	*h* = −13→13
Absorption correction: gaussian (CrysAlis PRO; Rigaku OD, 2018)	*k* = −49→52
*T*_min_ = 0.653, *T*_max_ = 1.000	*l* = −23→23
94929 measured reflections	

### Refinement

**Table:**

Refinement on *F*^2^	Primary atom site location: dual
Least-squares matrix: full	Hydrogen site location: mixed
*R*[*F*^2^ > 2σ(*F*^2^)] = 0.033	H atoms treated by a mixture of independent and constrained refinement
*wR*(*F*^2^) = 0.092	*w* = 1/[σ^2^(*F*_o_^2^) + (0.0481*P*)^2^ + 3.5049*P*] where *P* = (*F*_o_^2^ + 2*F*_c_^2^)/3
*S* = 1.04	(Δ/σ)_max_ = 0.003
17036 reflections	Δρ_max_ = 0.42 e Å^−^^3^
1079 parameters	Δρ_min_ = −0.55 e Å^−^^3^
0 restraints	

### Special details

**Table:**

Geometry. All esds (except the esd in the dihedral angle between two l.s. planes) are estimated using the full covariance matrix. The cell esds are taken into account individually in the estimation of esds in distances, angles and torsion angles; correlations between esds in cell parameters are only used when they are defined by crystal symmetry. An approximate (isotropic) treatment of cell esds is used for estimating esds involving l.s. planes.

### Fractional atomic coordinates and isotropic or equivalent isotropic displacement parameters (Å^2^)

**Table:**

	*x*	*y*	*z*	*U*_iso_*/*U*_eq_	
S1	0.86876 (3)	0.60239 (2)	0.45382 (2)	0.01933 (7)	
O5	0.66647 (18)	0.68394 (3)	0.41031 (6)	0.0653 (5)	
O6	0.63653 (11)	0.64495 (2)	0.48314 (5)	0.0298 (2)	
H6O	0.644 (2)	0.6616 (5)	0.5178 (12)	0.064 (7)*	
O7	1.05664 (8)	0.58755 (2)	0.57331 (5)	0.02348 (19)	
O8	1.25159 (9)	0.57343 (2)	0.55791 (5)	0.0250 (2)	
H8O	1.261 (2)	0.5646 (5)	0.6020 (12)	0.059 (6)*	
C73	0.57062 (11)	0.63409 (3)	0.30175 (6)	0.0189 (2)	
H73	0.510504	0.650346	0.296861	0.023*	
C74	0.56910 (12)	0.61279 (3)	0.24561 (7)	0.0208 (2)	
H74	0.507892	0.614647	0.203416	0.025*	
C75	0.65935 (13)	0.58870 (3)	0.25272 (7)	0.0231 (3)	
H75	0.659019	0.574453	0.215058	0.028*	
C76	0.75002 (12)	0.58580 (3)	0.31576 (7)	0.0227 (3)	
H76	0.809779	0.569467	0.320215	0.027*	
C77	0.75263 (11)	0.60713 (3)	0.37271 (6)	0.0180 (2)	
C78	0.66130 (11)	0.63139 (3)	0.36547 (6)	0.0161 (2)	
C79	0.65763 (12)	0.65610 (3)	0.42226 (6)	0.0198 (2)	
C80	1.01153 (11)	0.61285 (3)	0.42513 (6)	0.0167 (2)	
C81	1.01083 (13)	0.62905 (3)	0.36024 (7)	0.0234 (3)	
H81	0.933478	0.633723	0.329907	0.028*	
C82	1.12341 (14)	0.63818 (4)	0.34059 (8)	0.0297 (3)	
H82	1.120595	0.648892	0.297281	0.036*	
C83	1.24037 (13)	0.63157 (4)	0.38468 (8)	0.0294 (3)	
H83	1.315681	0.638162	0.371782	0.035*	
C84	1.24298 (12)	0.61502 (3)	0.44811 (7)	0.0232 (3)	
H84	1.321038	0.610079	0.477425	0.028*	
C85	1.13031 (11)	0.60555 (3)	0.46905 (6)	0.0168 (2)	
C86	1.14015 (11)	0.58810 (3)	0.53863 (6)	0.0170 (2)	
S2	0.36308 (3)	0.60471 (2)	0.95618 (2)	0.01877 (7)	
O9	0.56354 (8)	0.58971 (2)	1.06759 (4)	0.02008 (18)	
O10	0.75481 (8)	0.57505 (2)	1.04690 (5)	0.02276 (19)	
H10O	0.766 (2)	0.5668 (5)	1.0924 (12)	0.060 (6)*	
O11	0.12548 (13)	0.68518 (2)	0.90397 (5)	0.0423 (3)	
O12	0.15116 (10)	0.64745 (2)	0.98809 (5)	0.0254 (2)	
H12O	0.1545 (18)	0.6638 (5)	1.0188 (11)	0.045 (5)*	
C87	0.48876 (12)	0.62911 (3)	0.85303 (6)	0.0184 (2)	
H87	0.408551	0.634762	0.827278	0.022*	
C88	0.59558 (13)	0.63654 (3)	0.82546 (7)	0.0231 (3)	
H88	0.586377	0.647289	0.781755	0.028*	
C89	0.71638 (13)	0.62809 (3)	0.86246 (7)	0.0253 (3)	
H89	0.787995	0.632986	0.843699	0.030*	
C90	0.72868 (12)	0.61226 (3)	0.92771 (7)	0.0213 (2)	
H90	0.809347	0.606453	0.952545	0.026*	
C91	0.62200 (11)	0.60484 (3)	0.95702 (6)	0.0154 (2)	
C92	0.49939 (11)	0.61319 (3)	0.91922 (6)	0.0151 (2)	
C93	0.64177 (11)	0.58925 (3)	1.02911 (6)	0.0160 (2)	
C94	0.24038 (11)	0.60750 (3)	0.87723 (6)	0.0179 (2)	
C95	0.23738 (12)	0.58484 (3)	0.82279 (7)	0.0225 (3)	
H95	0.297462	0.568554	0.828932	0.027*	
C96	0.14576 (12)	0.58636 (3)	0.75969 (7)	0.0234 (3)	
H96	0.145014	0.571214	0.723745	0.028*	
C97	0.05526 (12)	0.61046 (3)	0.75020 (7)	0.0223 (3)	
H97	−0.006374	0.611467	0.708014	0.027*	
C98	0.05714 (11)	0.63302 (3)	0.80384 (6)	0.0194 (2)	
H98	−0.003174	0.649254	0.797145	0.023*	
C99	0.14835 (11)	0.63174 (3)	0.86788 (6)	0.0164 (2)	
C100	0.14237 (11)	0.65763 (3)	0.92171 (6)	0.0181 (2)	
P1	0.45306 (3)	0.53046 (2)	0.69428 (2)	0.01230 (6)	
O1	0.32078 (8)	0.54411 (2)	0.67897 (4)	0.01763 (17)	
C1	0.61145 (11)	0.53439 (3)	0.83070 (6)	0.0167 (2)	
H1	0.656091	0.550177	0.811734	0.020*	
C2	0.65061 (12)	0.52494 (3)	0.90237 (6)	0.0192 (2)	
H2	0.721498	0.534351	0.931197	0.023*	
C3	0.58344 (12)	0.50146 (3)	0.93049 (6)	0.0194 (2)	
H3	0.610599	0.494813	0.977939	0.023*	
C4	0.47584 (12)	0.48781 (3)	0.88817 (7)	0.0203 (2)	
H4	0.430003	0.472469	0.907717	0.024*	
C5	0.43666 (11)	0.49703 (3)	0.81683 (6)	0.0176 (2)	
H5	0.364754	0.487840	0.788474	0.021*	
C6	0.50568 (11)	0.52020 (3)	0.78760 (6)	0.0136 (2)	
C7	0.53838 (12)	0.46861 (3)	0.66991 (7)	0.0198 (2)	
H7	0.588550	0.469321	0.716034	0.024*	
C8	0.54019 (13)	0.44169 (3)	0.62722 (8)	0.0254 (3)	
H8	0.592086	0.424523	0.644795	0.030*	
C9	0.46540 (14)	0.44035 (3)	0.55898 (8)	0.0306 (3)	
H9	0.466881	0.422331	0.530569	0.037*	
C10	0.38826 (17)	0.46583 (4)	0.53291 (8)	0.0403 (4)	
H10	0.337265	0.464822	0.487039	0.048*	
C11	0.38629 (15)	0.49291 (4)	0.57465 (7)	0.0312 (3)	
H11	0.334659	0.510043	0.556555	0.037*	
C12	0.46162 (11)	0.49447 (3)	0.64372 (6)	0.0160 (2)	
C13	0.68681 (11)	0.54783 (3)	0.66017 (6)	0.0168 (2)	
H13	0.710047	0.526522	0.666794	0.020*	
C14	0.77099 (12)	0.56959 (3)	0.63990 (6)	0.0203 (2)	
H14	0.850763	0.562941	0.633391	0.024*	
C15	0.73532 (12)	0.60136 (3)	0.62942 (7)	0.0223 (3)	
H15	0.790696	0.615862	0.614766	0.027*	
C16	0.61775 (13)	0.61152 (3)	0.64067 (7)	0.0228 (3)	
H16	0.595084	0.632870	0.634256	0.027*	
C17	0.53354 (12)	0.58992 (3)	0.66151 (6)	0.0187 (2)	
H17	0.454903	0.596820	0.669357	0.022*	
C18	0.56734 (11)	0.55785 (3)	0.67064 (6)	0.0141 (2)	
P2	−0.04151 (3)	0.53337 (2)	0.18925 (2)	0.01230 (6)	
O2	−0.17363 (8)	0.54704 (2)	0.17137 (4)	0.01732 (17)	
C19	0.11406 (11)	0.53774 (3)	0.32684 (6)	0.0167 (2)	
H19	0.160010	0.553259	0.307909	0.020*	
C20	0.15104 (12)	0.52863 (3)	0.39889 (6)	0.0194 (2)	
H20	0.221743	0.538005	0.427976	0.023*	
C21	0.08202 (12)	0.50557 (3)	0.42697 (6)	0.0205 (2)	
H21	0.107516	0.499240	0.474765	0.025*	
C22	−0.02503 (12)	0.49184 (3)	0.38417 (7)	0.0211 (2)	
H22	−0.071898	0.476702	0.403634	0.025*	
C23	−0.06198 (11)	0.50071 (3)	0.31239 (6)	0.0176 (2)	
H23	−0.133366	0.491457	0.283687	0.021*	
C24	0.00844 (11)	0.52362 (3)	0.28324 (6)	0.0139 (2)	
C25	0.04771 (12)	0.47170 (3)	0.16729 (6)	0.0187 (2)	
H25	0.099716	0.473162	0.212801	0.022*	
C26	0.04960 (12)	0.44426 (3)	0.12632 (7)	0.0226 (3)	
H26	0.103258	0.427486	0.144451	0.027*	
C27	−0.02776 (13)	0.44180 (3)	0.05889 (7)	0.0257 (3)	
H27	−0.025983	0.423458	0.031542	0.031*	
C28	−0.10793 (15)	0.46669 (4)	0.03213 (8)	0.0345 (3)	
H28	−0.160726	0.464959	−0.013102	0.041*	
C29	−0.11000 (14)	0.49422 (3)	0.07241 (7)	0.0280 (3)	
H29	−0.163834	0.510912	0.053961	0.034*	
C30	−0.03175 (11)	0.49697 (3)	0.14038 (6)	0.0151 (2)	
C31	0.03988 (12)	0.59295 (3)	0.15930 (7)	0.0192 (2)	
H31	−0.039378	0.599663	0.166545	0.023*	
C32	0.12523 (13)	0.61484 (3)	0.14071 (7)	0.0234 (3)	
H32	0.102725	0.636236	0.135335	0.028*	
C33	0.24365 (12)	0.60499 (3)	0.13014 (7)	0.0212 (3)	
H33	0.299701	0.619741	0.117004	0.025*	
C34	0.27898 (11)	0.57320 (3)	0.13908 (6)	0.0193 (2)	
H34	0.359108	0.566710	0.132823	0.023*	
C35	0.19411 (11)	0.55108 (3)	0.15742 (6)	0.0161 (2)	
H35	0.217465	0.529756	0.163287	0.019*	
C36	0.07374 (11)	0.56081 (3)	0.16705 (6)	0.0142 (2)	
P3	0.09805 (3)	0.71259 (2)	0.12227 (2)	0.01599 (7)	
O3	0.17981 (8)	0.69166 (2)	0.08605 (5)	0.02155 (18)	
C37	−0.08417 (12)	0.68424 (3)	0.19179 (7)	0.0244 (3)	
H37	−0.030988	0.688982	0.235659	0.029*	
C38	−0.19660 (13)	0.66742 (3)	0.19036 (8)	0.0315 (3)	
H38	−0.218007	0.660839	0.233340	0.038*	
C39	−0.27655 (13)	0.66046 (3)	0.12557 (9)	0.0312 (3)	
H39	−0.351472	0.649149	0.124875	0.037*	
C40	−0.24498 (14)	0.67032 (3)	0.06181 (8)	0.0323 (3)	
H40	−0.299242	0.665798	0.018164	0.039*	
C41	−0.13297 (14)	0.68691 (3)	0.06245 (7)	0.0274 (3)	
H41	−0.112158	0.693373	0.019234	0.033*	
C42	−0.05124 (12)	0.69397 (3)	0.12764 (7)	0.0185 (2)	
C43	−0.03715 (12)	0.76945 (3)	0.08915 (8)	0.0248 (3)	
H43	−0.081000	0.764003	0.125165	0.030*	
C44	−0.06608 (13)	0.79759 (3)	0.05104 (8)	0.0299 (3)	
H44	−0.128479	0.811110	0.061853	0.036*	
C45	−0.00170 (14)	0.80552 (3)	−0.00327 (8)	0.0316 (3)	
H45	−0.021237	0.824361	−0.028957	0.038*	
C46	0.09146 (14)	0.78551 (3)	−0.01936 (8)	0.0305 (3)	
H46	0.134234	0.790946	−0.055853	0.037*	
C47	0.12140 (12)	0.75730 (3)	0.01881 (7)	0.0239 (3)	
H47	0.183816	0.743850	0.007752	0.029*	
C48	0.05757 (12)	0.74923 (3)	0.07371 (7)	0.0193 (2)	
C49	0.29856 (12)	0.70791 (3)	0.23785 (7)	0.0233 (3)	
H49	0.334641	0.694746	0.207831	0.028*	
C50	0.36378 (13)	0.71402 (3)	0.30779 (8)	0.0293 (3)	
H50	0.443912	0.705170	0.324258	0.035*	
C51	0.30930 (14)	0.73330 (3)	0.35291 (8)	0.0312 (3)	
H51	0.352266	0.736994	0.399933	0.037*	
C52	0.19116 (14)	0.74706 (3)	0.32820 (8)	0.0308 (3)	
H52	0.155341	0.760138	0.358497	0.037*	
C53	0.12620 (13)	0.74136 (3)	0.25837 (7)	0.0253 (3)	
H53	0.047151	0.750767	0.241795	0.030*	
C54	0.17919 (12)	0.72151 (3)	0.21276 (7)	0.0188 (2)	
P4	0.59434 (3)	0.71241 (2)	0.62054 (2)	0.01558 (7)	
O4	0.65852 (8)	0.68597 (2)	0.58832 (4)	0.01947 (18)	
C55	0.36994 (13)	0.67928 (3)	0.59742 (8)	0.0282 (3)	
H55	0.389594	0.674863	0.552729	0.034*	
C56	0.26067 (15)	0.66662 (4)	0.61513 (9)	0.0361 (3)	
H56	0.206716	0.653874	0.582208	0.043*	
C57	0.23188 (14)	0.67296 (4)	0.68190 (9)	0.0357 (3)	
H57	0.158804	0.664312	0.693791	0.043*	
C58	0.31085 (14)	0.69200 (4)	0.73083 (8)	0.0324 (3)	
H58	0.290945	0.696173	0.775564	0.039*	
C59	0.42035 (13)	0.70500 (3)	0.71334 (7)	0.0248 (3)	
H59	0.473371	0.717929	0.746290	0.030*	
C60	0.45058 (12)	0.69863 (3)	0.64644 (7)	0.0190 (2)	
C61	0.77928 (13)	0.70565 (3)	0.74264 (7)	0.0256 (3)	
H61	0.789190	0.685356	0.724829	0.031*	
C62	0.85001 (14)	0.71467 (4)	0.80939 (8)	0.0328 (3)	
H62	0.907827	0.700498	0.835864	0.039*	
C63	0.83457 (14)	0.74466 (4)	0.83647 (7)	0.0310 (3)	
H63	0.880548	0.750466	0.881635	0.037*	
C64	0.75106 (15)	0.76599 (4)	0.79662 (8)	0.0351 (3)	
H64	0.741536	0.786242	0.814744	0.042*	
C65	0.68114 (14)	0.75731 (3)	0.72948 (8)	0.0313 (3)	
H65	0.625732	0.771861	0.702523	0.038*	
C66	0.69371 (11)	0.72688 (3)	0.70238 (7)	0.0191 (2)	
C67	0.45982 (12)	0.76745 (3)	0.56674 (7)	0.0231 (3)	
H67	0.417547	0.765712	0.604988	0.028*	
C68	0.43043 (13)	0.79226 (3)	0.51757 (8)	0.0275 (3)	
H68	0.369387	0.807282	0.523296	0.033*	
C69	0.49220 (13)	0.79458 (3)	0.46002 (8)	0.0283 (3)	
H69	0.472196	0.811139	0.427055	0.034*	
C70	0.58383 (13)	0.77230 (3)	0.45135 (7)	0.0261 (3)	
H70	0.624512	0.773921	0.412430	0.031*	
C71	0.61497 (12)	0.74760 (3)	0.50053 (7)	0.0207 (2)	
H71	0.676790	0.732791	0.494812	0.025*	
C72	0.55292 (11)	0.74510 (3)	0.55873 (7)	0.0183 (2)	

### Atomic displacement parameters (Å^2^)

**Table:**

	*U*^11^	*U*^22^	*U*^33^	*U*^12^	*U*^13^	*U*^23^
S1	0.01503 (14)	0.02690 (16)	0.01590 (14)	0.00414 (11)	0.00267 (11)	0.00504 (11)
O5	0.1541 (15)	0.0195 (5)	0.0287 (6)	−0.0136 (7)	0.0334 (8)	−0.0030 (4)
O6	0.0519 (6)	0.0196 (5)	0.0228 (5)	−0.0019 (4)	0.0189 (4)	−0.0022 (4)
O7	0.0198 (4)	0.0312 (5)	0.0197 (4)	0.0043 (4)	0.0047 (3)	0.0069 (4)
O8	0.0187 (4)	0.0351 (5)	0.0200 (4)	0.0091 (4)	0.0011 (3)	0.0089 (4)
C73	0.0162 (5)	0.0211 (6)	0.0188 (6)	0.0022 (5)	0.0022 (4)	0.0008 (5)
C74	0.0175 (6)	0.0274 (6)	0.0164 (6)	−0.0040 (5)	0.0004 (4)	−0.0018 (5)
C75	0.0251 (6)	0.0255 (6)	0.0194 (6)	−0.0024 (5)	0.0065 (5)	−0.0073 (5)
C76	0.0211 (6)	0.0232 (6)	0.0249 (6)	0.0056 (5)	0.0072 (5)	−0.0028 (5)
C77	0.0162 (5)	0.0206 (6)	0.0170 (5)	0.0017 (5)	0.0028 (4)	0.0014 (5)
C78	0.0160 (5)	0.0161 (5)	0.0164 (5)	0.0000 (4)	0.0037 (4)	0.0006 (4)
C79	0.0234 (6)	0.0185 (6)	0.0165 (6)	0.0024 (5)	0.0018 (5)	0.0007 (5)
C80	0.0186 (6)	0.0154 (5)	0.0164 (5)	0.0031 (4)	0.0042 (4)	0.0003 (4)
C81	0.0235 (6)	0.0277 (7)	0.0192 (6)	0.0062 (5)	0.0046 (5)	0.0069 (5)
C82	0.0310 (7)	0.0344 (7)	0.0262 (7)	0.0060 (6)	0.0116 (6)	0.0128 (6)
C83	0.0246 (7)	0.0337 (7)	0.0334 (7)	0.0006 (6)	0.0139 (6)	0.0093 (6)
C84	0.0177 (6)	0.0260 (6)	0.0260 (6)	0.0022 (5)	0.0044 (5)	0.0043 (5)
C85	0.0190 (6)	0.0149 (5)	0.0165 (5)	0.0018 (4)	0.0037 (4)	0.0003 (4)
C86	0.0165 (5)	0.0161 (5)	0.0168 (5)	0.0009 (4)	−0.0005 (4)	−0.0003 (4)
S2	0.01312 (13)	0.02878 (16)	0.01450 (13)	0.00328 (11)	0.00294 (10)	0.00385 (11)
O9	0.0180 (4)	0.0253 (4)	0.0169 (4)	0.0024 (3)	0.0033 (3)	0.0036 (3)
O10	0.0172 (4)	0.0317 (5)	0.0182 (4)	0.0088 (4)	0.0007 (3)	0.0046 (4)
O11	0.0794 (9)	0.0169 (5)	0.0236 (5)	−0.0017 (5)	−0.0069 (5)	0.0018 (4)
O12	0.0420 (6)	0.0184 (4)	0.0189 (4)	0.0065 (4)	0.0136 (4)	0.0013 (4)
C87	0.0184 (6)	0.0192 (6)	0.0168 (5)	0.0033 (5)	0.0016 (4)	0.0022 (4)
C88	0.0278 (7)	0.0233 (6)	0.0196 (6)	0.0017 (5)	0.0083 (5)	0.0060 (5)
C89	0.0212 (6)	0.0294 (7)	0.0281 (7)	0.0002 (5)	0.0118 (5)	0.0054 (5)
C90	0.0159 (6)	0.0238 (6)	0.0246 (6)	0.0019 (5)	0.0050 (5)	0.0021 (5)
C91	0.0152 (5)	0.0150 (5)	0.0157 (5)	0.0013 (4)	0.0027 (4)	−0.0007 (4)
C92	0.0166 (5)	0.0137 (5)	0.0152 (5)	0.0014 (4)	0.0037 (4)	−0.0008 (4)
C93	0.0154 (5)	0.0150 (5)	0.0162 (5)	0.0005 (4)	−0.0003 (4)	−0.0012 (4)
C94	0.0138 (5)	0.0237 (6)	0.0167 (5)	−0.0001 (5)	0.0037 (4)	0.0023 (5)
C95	0.0187 (6)	0.0264 (6)	0.0236 (6)	0.0027 (5)	0.0073 (5)	−0.0017 (5)
C96	0.0227 (6)	0.0292 (7)	0.0193 (6)	−0.0042 (5)	0.0063 (5)	−0.0050 (5)
C97	0.0177 (6)	0.0309 (7)	0.0167 (6)	−0.0058 (5)	−0.0003 (5)	−0.0003 (5)
C98	0.0151 (5)	0.0234 (6)	0.0193 (6)	−0.0011 (5)	0.0024 (5)	0.0035 (5)
C99	0.0142 (5)	0.0186 (6)	0.0166 (5)	−0.0027 (4)	0.0036 (4)	0.0027 (4)
C100	0.0155 (5)	0.0191 (6)	0.0179 (6)	−0.0015 (4)	−0.0007 (4)	0.0024 (5)
P1	0.01150 (13)	0.01351 (13)	0.01110 (13)	0.00026 (10)	0.00034 (10)	0.00070 (10)
O1	0.0128 (4)	0.0217 (4)	0.0176 (4)	0.0025 (3)	0.0013 (3)	0.0031 (3)
C1	0.0172 (6)	0.0182 (5)	0.0144 (5)	−0.0027 (4)	0.0023 (4)	−0.0004 (4)
C2	0.0172 (6)	0.0259 (6)	0.0133 (5)	−0.0021 (5)	−0.0001 (4)	−0.0022 (5)
C3	0.0210 (6)	0.0254 (6)	0.0120 (5)	0.0037 (5)	0.0035 (4)	0.0022 (5)
C4	0.0228 (6)	0.0210 (6)	0.0180 (6)	−0.0027 (5)	0.0062 (5)	0.0037 (5)
C5	0.0172 (5)	0.0188 (6)	0.0161 (5)	−0.0027 (5)	0.0014 (4)	−0.0002 (4)
C6	0.0146 (5)	0.0137 (5)	0.0125 (5)	0.0017 (4)	0.0024 (4)	−0.0003 (4)
C7	0.0202 (6)	0.0193 (6)	0.0196 (6)	0.0003 (5)	0.0030 (5)	−0.0023 (5)
C8	0.0257 (7)	0.0180 (6)	0.0349 (7)	−0.0006 (5)	0.0118 (6)	−0.0047 (5)
C9	0.0329 (7)	0.0278 (7)	0.0336 (7)	−0.0083 (6)	0.0124 (6)	−0.0168 (6)
C10	0.0489 (10)	0.0429 (9)	0.0232 (7)	0.0014 (7)	−0.0069 (7)	−0.0169 (6)
C11	0.0382 (8)	0.0311 (7)	0.0190 (6)	0.0060 (6)	−0.0069 (6)	−0.0056 (5)
C12	0.0155 (5)	0.0173 (5)	0.0149 (5)	−0.0032 (4)	0.0026 (4)	−0.0016 (4)
C13	0.0167 (5)	0.0175 (5)	0.0153 (5)	0.0000 (4)	0.0015 (4)	−0.0014 (4)
C14	0.0156 (5)	0.0280 (6)	0.0172 (6)	−0.0037 (5)	0.0032 (4)	−0.0022 (5)
C15	0.0227 (6)	0.0253 (6)	0.0175 (6)	−0.0089 (5)	0.0009 (5)	0.0035 (5)
C16	0.0272 (7)	0.0166 (6)	0.0232 (6)	−0.0022 (5)	0.0016 (5)	0.0043 (5)
C17	0.0186 (6)	0.0174 (6)	0.0195 (6)	0.0016 (5)	0.0024 (5)	0.0015 (4)
C18	0.0155 (5)	0.0162 (5)	0.0096 (5)	−0.0013 (4)	0.0000 (4)	0.0002 (4)
P2	0.01140 (13)	0.01358 (13)	0.01122 (13)	0.00096 (10)	0.00054 (10)	0.00039 (10)
O2	0.0131 (4)	0.0211 (4)	0.0170 (4)	0.0036 (3)	0.0013 (3)	0.0014 (3)
C19	0.0175 (6)	0.0175 (5)	0.0151 (5)	−0.0020 (4)	0.0028 (4)	−0.0006 (4)
C20	0.0178 (6)	0.0253 (6)	0.0136 (5)	−0.0021 (5)	−0.0002 (4)	−0.0030 (5)
C21	0.0222 (6)	0.0265 (6)	0.0126 (5)	0.0023 (5)	0.0031 (5)	0.0018 (5)
C22	0.0235 (6)	0.0232 (6)	0.0171 (6)	−0.0031 (5)	0.0058 (5)	0.0032 (5)
C23	0.0169 (5)	0.0193 (6)	0.0159 (5)	−0.0023 (5)	0.0019 (4)	−0.0003 (4)
C24	0.0148 (5)	0.0140 (5)	0.0129 (5)	0.0023 (4)	0.0027 (4)	−0.0005 (4)
C25	0.0184 (6)	0.0192 (6)	0.0176 (6)	0.0011 (5)	0.0013 (5)	−0.0015 (5)
C26	0.0228 (6)	0.0175 (6)	0.0288 (7)	0.0017 (5)	0.0085 (5)	−0.0025 (5)
C27	0.0255 (7)	0.0243 (6)	0.0288 (7)	−0.0048 (5)	0.0093 (5)	−0.0125 (5)
C28	0.0375 (8)	0.0377 (8)	0.0231 (7)	0.0042 (7)	−0.0066 (6)	−0.0143 (6)
C29	0.0323 (7)	0.0288 (7)	0.0183 (6)	0.0086 (6)	−0.0062 (5)	−0.0050 (5)
C30	0.0142 (5)	0.0166 (5)	0.0146 (5)	−0.0010 (4)	0.0030 (4)	−0.0014 (4)
C31	0.0189 (6)	0.0175 (6)	0.0215 (6)	0.0031 (5)	0.0046 (5)	0.0019 (5)
C32	0.0275 (7)	0.0154 (6)	0.0273 (6)	0.0013 (5)	0.0051 (5)	0.0048 (5)
C33	0.0218 (6)	0.0221 (6)	0.0189 (6)	−0.0056 (5)	0.0020 (5)	0.0043 (5)
C34	0.0155 (5)	0.0247 (6)	0.0176 (6)	−0.0018 (5)	0.0027 (4)	−0.0016 (5)
C35	0.0169 (6)	0.0154 (5)	0.0153 (5)	0.0010 (4)	0.0012 (4)	−0.0010 (4)
C36	0.0157 (5)	0.0158 (5)	0.0101 (5)	0.0001 (4)	0.0002 (4)	−0.0002 (4)
P3	0.01686 (14)	0.01392 (14)	0.01790 (14)	0.00187 (11)	0.00514 (11)	−0.00059 (11)
O3	0.0249 (4)	0.0197 (4)	0.0215 (4)	0.0058 (4)	0.0082 (4)	−0.0020 (3)
C37	0.0198 (6)	0.0265 (6)	0.0257 (6)	−0.0007 (5)	0.0020 (5)	0.0058 (5)
C38	0.0247 (7)	0.0321 (7)	0.0380 (8)	−0.0025 (6)	0.0066 (6)	0.0114 (6)
C39	0.0210 (6)	0.0197 (6)	0.0507 (9)	−0.0035 (5)	0.0018 (6)	0.0018 (6)
C40	0.0296 (7)	0.0229 (7)	0.0390 (8)	−0.0050 (6)	−0.0060 (6)	−0.0052 (6)
C41	0.0332 (7)	0.0230 (6)	0.0245 (6)	−0.0046 (6)	0.0020 (5)	−0.0029 (5)
C42	0.0184 (6)	0.0124 (5)	0.0241 (6)	0.0016 (4)	0.0032 (5)	−0.0001 (4)
C43	0.0209 (6)	0.0217 (6)	0.0337 (7)	0.0023 (5)	0.0102 (5)	0.0037 (5)
C44	0.0231 (6)	0.0202 (6)	0.0472 (8)	0.0053 (5)	0.0085 (6)	0.0047 (6)
C45	0.0305 (7)	0.0203 (6)	0.0432 (8)	0.0004 (6)	0.0054 (6)	0.0114 (6)
C46	0.0315 (7)	0.0281 (7)	0.0339 (7)	−0.0005 (6)	0.0109 (6)	0.0091 (6)
C47	0.0231 (6)	0.0227 (6)	0.0273 (6)	0.0015 (5)	0.0083 (5)	0.0032 (5)
C48	0.0182 (6)	0.0162 (6)	0.0234 (6)	−0.0002 (5)	0.0039 (5)	0.0014 (5)
C49	0.0202 (6)	0.0230 (6)	0.0270 (7)	0.0017 (5)	0.0051 (5)	−0.0018 (5)
C50	0.0215 (6)	0.0308 (7)	0.0325 (7)	0.0011 (5)	−0.0021 (5)	0.0002 (6)
C51	0.0353 (8)	0.0301 (7)	0.0249 (7)	−0.0059 (6)	−0.0019 (6)	−0.0052 (6)
C52	0.0379 (8)	0.0267 (7)	0.0279 (7)	0.0008 (6)	0.0065 (6)	−0.0095 (6)
C53	0.0257 (6)	0.0234 (6)	0.0266 (7)	0.0039 (5)	0.0046 (5)	−0.0051 (5)
C54	0.0185 (6)	0.0165 (5)	0.0215 (6)	−0.0015 (5)	0.0040 (5)	−0.0014 (5)
P4	0.01651 (14)	0.01290 (14)	0.01749 (14)	0.00079 (11)	0.00375 (11)	−0.00068 (11)
O4	0.0241 (4)	0.0156 (4)	0.0196 (4)	0.0037 (3)	0.0063 (3)	−0.0012 (3)
C55	0.0279 (7)	0.0244 (7)	0.0328 (7)	−0.0063 (5)	0.0073 (6)	−0.0046 (5)
C56	0.0295 (7)	0.0259 (7)	0.0530 (9)	−0.0089 (6)	0.0084 (7)	−0.0013 (7)
C57	0.0253 (7)	0.0294 (7)	0.0562 (10)	0.0005 (6)	0.0170 (7)	0.0153 (7)
C58	0.0294 (7)	0.0373 (8)	0.0344 (7)	0.0095 (6)	0.0157 (6)	0.0123 (6)
C59	0.0236 (6)	0.0267 (7)	0.0251 (6)	0.0043 (5)	0.0071 (5)	0.0026 (5)
C60	0.0193 (6)	0.0143 (5)	0.0239 (6)	0.0025 (5)	0.0055 (5)	0.0036 (5)
C61	0.0236 (6)	0.0248 (6)	0.0268 (7)	0.0015 (5)	0.0015 (5)	−0.0020 (5)
C62	0.0287 (7)	0.0372 (8)	0.0288 (7)	0.0001 (6)	−0.0033 (6)	0.0010 (6)
C63	0.0282 (7)	0.0435 (8)	0.0216 (6)	−0.0135 (6)	0.0058 (5)	−0.0076 (6)
C64	0.0381 (8)	0.0325 (8)	0.0342 (8)	−0.0052 (6)	0.0061 (6)	−0.0163 (6)
C65	0.0340 (7)	0.0241 (7)	0.0330 (7)	0.0036 (6)	0.0001 (6)	−0.0085 (6)
C66	0.0173 (6)	0.0205 (6)	0.0202 (6)	−0.0024 (5)	0.0050 (5)	−0.0025 (5)
C67	0.0192 (6)	0.0202 (6)	0.0309 (7)	0.0021 (5)	0.0072 (5)	0.0029 (5)
C68	0.0197 (6)	0.0213 (6)	0.0414 (8)	0.0045 (5)	0.0056 (6)	0.0067 (6)
C69	0.0258 (7)	0.0227 (6)	0.0352 (7)	0.0012 (5)	0.0028 (6)	0.0117 (6)
C70	0.0260 (7)	0.0248 (6)	0.0282 (7)	−0.0024 (5)	0.0074 (5)	0.0056 (5)
C71	0.0189 (6)	0.0181 (6)	0.0255 (6)	−0.0010 (5)	0.0052 (5)	0.0006 (5)
C72	0.0164 (6)	0.0153 (5)	0.0226 (6)	−0.0020 (4)	0.0024 (5)	0.0009 (5)

### Geometric parameters (Å, º)

**Table:**

S1—C77	1.7839 (12)	C20—H20	0.9300
S1—C80	1.7764 (12)	C20—C21	1.3866 (18)
O5—C79	1.1978 (17)	C21—H21	0.9300
O6—H6O	0.95 (2)	C21—C22	1.3902 (18)
O6—C79	1.3043 (15)	C22—H22	0.9300
O7—C86	1.2104 (15)	C22—C23	1.3880 (17)
O8—H8O	0.90 (2)	C23—H23	0.9300
O8—C86	1.3279 (15)	C23—C24	1.4015 (16)
C73—H73	0.9300	C25—H25	0.9300
C73—C74	1.3862 (17)	C25—C26	1.3910 (17)
C73—C78	1.3942 (17)	C25—C30	1.3914 (17)
C74—H74	0.9300	C26—H26	0.9300
C74—C75	1.3866 (19)	C26—C27	1.3799 (19)
C75—H75	0.9300	C27—H27	0.9300
C75—C76	1.3854 (19)	C27—C28	1.383 (2)
C76—H76	0.9300	C28—H28	0.9300
C76—C77	1.3975 (17)	C28—C29	1.3871 (19)
C77—C78	1.3996 (16)	C29—H29	0.9300
C78—C79	1.4998 (16)	C29—C30	1.3933 (17)
C80—C81	1.4024 (16)	C31—H31	0.9300
C80—C85	1.4077 (17)	C31—C32	1.3898 (18)
C81—H81	0.9300	C31—C36	1.3969 (16)
C81—C82	1.3847 (19)	C32—H32	0.9300
C82—H82	0.9300	C32—C33	1.3857 (19)
C82—C83	1.388 (2)	C33—H33	0.9300
C83—H83	0.9300	C33—C34	1.3883 (18)
C83—C84	1.3826 (18)	C34—H34	0.9300
C84—H84	0.9300	C34—C35	1.3907 (17)
C84—C85	1.4004 (17)	C35—H35	0.9300
C85—C86	1.4921 (16)	C35—C36	1.3983 (16)
S2—C92	1.7760 (12)	P3—O3	1.4991 (9)
S2—C94	1.7863 (12)	P3—C42	1.8004 (13)
O9—C93	1.2147 (15)	P3—C48	1.8001 (12)
O10—H10O	0.91 (2)	P3—C54	1.7989 (13)
O10—C93	1.3309 (14)	C37—H37	0.9300
O11—C100	1.2075 (16)	C37—C38	1.3912 (19)
O12—H12O	0.89 (2)	C37—C42	1.3915 (17)
O12—C100	1.3126 (15)	C38—H38	0.9300
C87—H87	0.9300	C38—C39	1.380 (2)
C87—C88	1.3842 (17)	C39—H39	0.9300
C87—C92	1.4042 (16)	C39—C40	1.380 (2)
C88—H88	0.9300	C40—H40	0.9300
C88—C89	1.3887 (19)	C40—C41	1.385 (2)
C89—H89	0.9300	C41—H41	0.9300
C89—C90	1.3853 (18)	C41—C42	1.3949 (18)
C90—H90	0.9300	C43—H43	0.9300
C90—C91	1.4008 (16)	C43—C44	1.3865 (19)
C91—C92	1.4087 (16)	C43—C48	1.3973 (17)
C91—C93	1.4900 (16)	C44—H44	0.9300
C94—C95	1.3980 (17)	C44—C45	1.388 (2)
C94—C99	1.4031 (17)	C45—H45	0.9300
C95—H95	0.9300	C45—C46	1.384 (2)
C95—C96	1.3879 (18)	C46—H46	0.9300
C96—H96	0.9300	C46—C47	1.3911 (18)
C96—C97	1.3876 (19)	C47—H47	0.9300
C97—H97	0.9300	C47—C48	1.3943 (17)
C97—C98	1.3856 (18)	C49—H49	0.9300
C98—H98	0.9300	C49—C50	1.3919 (19)
C98—C99	1.3979 (16)	C49—C54	1.3941 (18)
C99—C100	1.4997 (17)	C50—H50	0.9300
P1—O1	1.5018 (8)	C50—C51	1.387 (2)
P1—C6	1.7961 (11)	C51—H51	0.9300
P1—C12	1.8003 (12)	C51—C52	1.385 (2)
P1—C18	1.7994 (12)	C52—H52	0.9300
C1—H1	0.9300	C52—C53	1.3863 (19)
C1—C2	1.3952 (16)	C53—H53	0.9300
C1—C6	1.3909 (16)	C53—C54	1.3987 (17)
C2—H2	0.9300	P4—O4	1.4975 (8)
C2—C3	1.3874 (18)	P4—C60	1.8014 (13)
C3—H3	0.9300	P4—C66	1.8010 (13)
C3—C4	1.3890 (18)	P4—C72	1.8005 (12)
C4—H4	0.9300	C55—H55	0.9300
C4—C5	1.3868 (17)	C55—C56	1.386 (2)
C5—H5	0.9300	C55—C60	1.3955 (18)
C5—C6	1.4007 (16)	C56—H56	0.9300
C7—H7	0.9300	C56—C57	1.385 (2)
C7—C8	1.3915 (17)	C57—H57	0.9300
C7—C12	1.3920 (17)	C57—C58	1.378 (2)
C8—H8	0.9300	C58—H58	0.9300
C8—C9	1.379 (2)	C58—C59	1.3917 (19)
C9—H9	0.9300	C59—H59	0.9300
C9—C10	1.381 (2)	C59—C60	1.3937 (18)
C10—H10	0.9300	C61—H61	0.9300
C10—C11	1.387 (2)	C61—C62	1.3899 (19)
C11—H11	0.9300	C61—C66	1.3915 (18)
C11—C12	1.3941 (17)	C62—H62	0.9300
C13—H13	0.9300	C62—C63	1.381 (2)
C13—C14	1.3901 (17)	C63—H63	0.9300
C13—C18	1.3979 (16)	C63—C64	1.379 (2)
C14—H14	0.9300	C64—H64	0.9300
C14—C15	1.3902 (18)	C64—C65	1.389 (2)
C15—H15	0.9300	C65—H65	0.9300
C15—C16	1.3854 (19)	C65—C66	1.3929 (18)
C16—H16	0.9300	C67—H67	0.9300
C16—C17	1.3902 (17)	C67—C68	1.3902 (18)
C17—H17	0.9300	C67—C72	1.3991 (17)
C17—C18	1.3956 (16)	C68—H68	0.9300
P2—O2	1.5014 (8)	C68—C69	1.386 (2)
P2—C24	1.7997 (11)	C69—H69	0.9300
P2—C30	1.7998 (12)	C69—C70	1.389 (2)
P2—C36	1.7980 (12)	C70—H70	0.9300
C19—H19	0.9300	C70—C71	1.3889 (18)
C19—C20	1.3956 (16)	C71—H71	0.9300
C19—C24	1.3927 (16)	C71—C72	1.3990 (17)
			
C80—S1—C77	101.78 (6)	C21—C22—H22	120.0
C79—O6—H6O	109.8 (14)	C23—C22—C21	120.02 (11)
C86—O8—H8O	111.7 (14)	C23—C22—H22	120.0
C74—C73—H73	119.7	C22—C23—H23	120.0
C74—C73—C78	120.70 (11)	C22—C23—C24	119.91 (11)
C78—C73—H73	119.7	C24—C23—H23	120.0
C73—C74—H74	120.2	C19—C24—P2	122.36 (9)
C73—C74—C75	119.62 (11)	C19—C24—C23	119.73 (10)
C75—C74—H74	120.2	C23—C24—P2	117.90 (9)
C74—C75—H75	119.9	C26—C25—H25	119.9
C76—C75—C74	120.21 (11)	C26—C25—C30	120.21 (11)
C76—C75—H75	119.9	C30—C25—H25	119.9
C75—C76—H76	119.6	C25—C26—H26	119.8
C75—C76—C77	120.71 (12)	C27—C26—C25	120.31 (12)
C77—C76—H76	119.6	C27—C26—H26	119.8
C76—C77—S1	119.72 (9)	C26—C27—H27	120.1
C76—C77—C78	118.98 (11)	C26—C27—C28	119.79 (12)
C78—C77—S1	121.28 (9)	C28—C27—H27	120.1
C73—C78—C77	119.77 (11)	C27—C28—H28	119.8
C73—C78—C79	116.79 (11)	C27—C28—C29	120.33 (13)
C77—C78—C79	123.40 (11)	C29—C28—H28	119.8
O5—C79—O6	123.45 (12)	C28—C29—H29	119.9
O5—C79—C78	121.67 (12)	C28—C29—C30	120.27 (13)
O6—C79—C78	114.76 (11)	C30—C29—H29	119.9
C81—C80—S1	122.19 (9)	C25—C30—P2	123.58 (9)
C81—C80—C85	117.98 (11)	C25—C30—C29	119.09 (11)
C85—C80—S1	119.81 (9)	C29—C30—P2	117.33 (9)
C80—C81—H81	119.5	C32—C31—H31	120.2
C82—C81—C80	121.10 (12)	C32—C31—C36	119.67 (11)
C82—C81—H81	119.5	C36—C31—H31	120.2
C81—C82—H82	119.6	C31—C32—H32	119.8
C81—C82—C83	120.85 (12)	C33—C32—C31	120.40 (12)
C83—C82—H82	119.6	C33—C32—H32	119.8
C82—C83—H83	120.6	C32—C33—H33	119.9
C84—C83—C82	118.84 (12)	C32—C33—C34	120.29 (11)
C84—C83—H83	120.6	C34—C33—H33	119.9
C83—C84—H84	119.4	C33—C34—H34	120.1
C83—C84—C85	121.26 (12)	C33—C34—C35	119.76 (11)
C85—C84—H84	119.4	C35—C34—H34	120.1
C80—C85—C86	121.64 (11)	C34—C35—H35	119.9
C84—C85—C80	119.94 (11)	C34—C35—C36	120.18 (11)
C84—C85—C86	118.41 (11)	C36—C35—H35	119.9
O7—C86—O8	124.24 (11)	C31—C36—P2	117.85 (9)
O7—C86—C85	123.93 (11)	C31—C36—C35	119.68 (11)
O8—C86—C85	111.83 (10)	C35—C36—P2	122.46 (9)
C92—S2—C94	100.52 (5)	O3—P3—C42	112.18 (5)
C93—O10—H10O	110.6 (14)	O3—P3—C48	111.75 (5)
C100—O12—H12O	111.0 (12)	O3—P3—C54	110.01 (6)
C88—C87—H87	119.4	C48—P3—C42	105.87 (6)
C88—C87—C92	121.16 (11)	C54—P3—C42	107.61 (6)
C92—C87—H87	119.4	C54—P3—C48	109.24 (6)
C87—C88—H88	119.7	C38—C37—H37	120.0
C87—C88—C89	120.59 (11)	C38—C37—C42	120.05 (13)
C89—C88—H88	119.7	C42—C37—H37	120.0
C88—C89—H89	120.5	C37—C38—H38	119.8
C90—C89—C88	119.06 (12)	C39—C38—C37	120.41 (13)
C90—C89—H89	120.5	C39—C38—H38	119.8
C89—C90—H90	119.4	C38—C39—H39	120.1
C89—C90—C91	121.30 (12)	C38—C39—C40	119.79 (13)
C91—C90—H90	119.4	C40—C39—H39	120.1
C90—C91—C92	119.64 (11)	C39—C40—H40	119.8
C90—C91—C93	118.82 (11)	C39—C40—C41	120.39 (13)
C92—C91—C93	121.50 (10)	C41—C40—H40	119.8
C87—C92—S2	121.33 (9)	C40—C41—H41	119.9
C87—C92—C91	118.24 (11)	C40—C41—C42	120.28 (13)
C91—C92—S2	120.40 (9)	C42—C41—H41	119.9
O9—C93—O10	123.83 (11)	C37—C42—P3	123.98 (10)
O9—C93—C91	123.56 (11)	C37—C42—C41	119.08 (12)
O10—C93—C91	112.61 (10)	C41—C42—P3	116.74 (10)
C95—C94—S2	118.49 (9)	C44—C43—H43	119.8
C95—C94—C99	119.21 (11)	C44—C43—C48	120.32 (12)
C99—C94—S2	122.30 (9)	C48—C43—H43	119.8
C94—C95—H95	119.6	C43—C44—H44	120.1
C96—C95—C94	120.76 (12)	C43—C44—C45	119.82 (13)
C96—C95—H95	119.6	C45—C44—H44	120.1
C95—C96—H96	120.0	C44—C45—H45	119.9
C97—C96—C95	120.05 (12)	C46—C45—C44	120.25 (13)
C97—C96—H96	120.0	C46—C45—H45	119.9
C96—C97—H97	120.1	C45—C46—H46	119.9
C98—C97—C96	119.71 (12)	C45—C46—C47	120.28 (13)
C98—C97—H97	120.1	C47—C46—H46	119.9
C97—C98—H98	119.5	C46—C47—H47	120.1
C97—C98—C99	120.98 (12)	C46—C47—C48	119.82 (12)
C99—C98—H98	119.5	C48—C47—H47	120.1
C94—C99—C100	124.32 (11)	C43—C48—P3	121.55 (10)
C98—C99—C94	119.28 (11)	C47—C48—P3	118.94 (9)
C98—C99—C100	116.38 (11)	C47—C48—C43	119.50 (12)
O11—C100—O12	123.80 (12)	C50—C49—H49	120.0
O11—C100—C99	121.95 (11)	C50—C49—C54	120.05 (12)
O12—C100—C99	114.18 (10)	C54—C49—H49	120.0
O1—P1—C6	112.35 (5)	C49—C50—H50	120.0
O1—P1—C12	111.32 (5)	C51—C50—C49	120.01 (13)
O1—P1—C18	111.73 (5)	C51—C50—H50	120.0
C6—P1—C12	106.40 (5)	C50—C51—H51	119.9
C6—P1—C18	107.57 (5)	C52—C51—C50	120.25 (13)
C18—P1—C12	107.14 (5)	C52—C51—H51	119.9
C2—C1—H1	120.0	C51—C52—H52	120.0
C6—C1—H1	120.0	C51—C52—C53	120.08 (13)
C6—C1—C2	120.03 (11)	C53—C52—H52	120.0
C1—C2—H2	120.1	C52—C53—H53	119.9
C3—C2—C1	119.77 (11)	C52—C53—C54	120.19 (13)
C3—C2—H2	120.1	C54—C53—H53	119.9
C2—C3—H3	119.8	C49—C54—P3	118.37 (9)
C2—C3—C4	120.38 (11)	C49—C54—C53	119.40 (12)
C4—C3—H3	119.8	C53—C54—P3	122.23 (10)
C3—C4—H4	119.9	O4—P4—C60	111.07 (5)
C5—C4—C3	120.12 (11)	O4—P4—C66	111.08 (5)
C5—C4—H4	119.9	O4—P4—C72	111.95 (5)
C4—C5—H5	120.1	C66—P4—C60	105.35 (6)
C4—C5—C6	119.81 (11)	C72—P4—C60	107.95 (6)
C6—C5—H5	120.1	C72—P4—C66	109.19 (6)
C1—C6—P1	122.24 (9)	C56—C55—H55	119.9
C1—C6—C5	119.86 (10)	C56—C55—C60	120.19 (13)
C5—C6—P1	117.90 (9)	C60—C55—H55	119.9
C8—C7—H7	119.9	C55—C56—H56	120.0
C8—C7—C12	120.14 (12)	C57—C56—C55	119.95 (14)
C12—C7—H7	119.9	C57—C56—H56	120.0
C7—C8—H8	119.9	C56—C57—H57	119.8
C9—C8—C7	120.28 (13)	C58—C57—C56	120.41 (13)
C9—C8—H8	119.9	C58—C57—H57	119.8
C8—C9—H9	120.1	C57—C58—H58	120.0
C8—C9—C10	119.85 (12)	C57—C58—C59	120.05 (14)
C10—C9—H9	120.1	C59—C58—H58	120.0
C9—C10—H10	119.8	C58—C59—H59	120.0
C9—C10—C11	120.47 (13)	C58—C59—C60	120.01 (13)
C11—C10—H10	119.8	C60—C59—H59	120.0
C10—C11—H11	120.0	C55—C60—P4	117.07 (10)
C10—C11—C12	120.08 (13)	C59—C60—P4	123.46 (10)
C12—C11—H11	120.0	C59—C60—C55	119.39 (12)
C7—C12—P1	123.65 (9)	C62—C61—H61	119.9
C7—C12—C11	119.17 (11)	C62—C61—C66	120.23 (13)
C11—C12—P1	117.18 (10)	C66—C61—H61	119.9
C14—C13—H13	119.9	C61—C62—H62	119.9
C14—C13—C18	120.22 (11)	C63—C62—C61	120.12 (14)
C18—C13—H13	119.9	C63—C62—H62	119.9
C13—C14—H14	120.2	C62—C63—H63	120.0
C13—C14—C15	119.66 (12)	C64—C63—C62	120.07 (13)
C15—C14—H14	120.2	C64—C63—H63	120.0
C14—C15—H15	119.8	C63—C64—H64	119.9
C16—C15—C14	120.38 (12)	C63—C64—C65	120.19 (13)
C16—C15—H15	119.8	C65—C64—H64	119.9
C15—C16—H16	119.9	C64—C65—H65	119.9
C15—C16—C17	120.22 (12)	C64—C65—C66	120.23 (14)
C17—C16—H16	119.9	C66—C65—H65	119.9
C16—C17—H17	120.1	C61—C66—P4	117.83 (10)
C16—C17—C18	119.82 (11)	C61—C66—C65	119.14 (12)
C18—C17—H17	120.1	C65—C66—P4	122.83 (10)
C13—C18—P1	121.99 (9)	C68—C67—H67	120.0
C17—C18—P1	118.34 (9)	C68—C67—C72	120.06 (12)
C17—C18—C13	119.66 (11)	C72—C67—H67	120.0
O2—P2—C24	113.00 (5)	C67—C68—H68	120.0
O2—P2—C30	110.92 (5)	C69—C68—C67	119.93 (12)
O2—P2—C36	111.25 (5)	C69—C68—H68	120.0
C24—P2—C30	106.18 (5)	C68—C69—H69	119.8
C36—P2—C24	107.09 (5)	C68—C69—C70	120.30 (12)
C36—P2—C30	108.12 (5)	C70—C69—H69	119.8
C20—C19—H19	120.0	C69—C70—H70	119.9
C24—C19—H19	120.0	C69—C70—C71	120.30 (12)
C24—C19—C20	120.09 (11)	C71—C70—H70	119.9
C19—C20—H20	120.1	C70—C71—H71	120.2
C21—C20—C19	119.78 (11)	C70—C71—C72	119.70 (12)
C21—C20—H20	120.1	C72—C71—H71	120.2
C20—C21—H21	119.8	C67—C72—P4	121.96 (10)
C20—C21—C22	120.45 (11)	C67—C72—C71	119.70 (11)
C22—C21—H21	119.8	C71—C72—P4	118.33 (9)

### Hydrogen-bond geometry (Å, º)

**Table:**

*D*—H···*A*	*D*—H	H···*A*	*D*···*A*	*D*—H···*A*
O6—H6*O*···O4	0.95 (2)	1.66 (2)	2.6070 (12)	171 (2)
O8—H8*O*···O1^i^	0.90 (2)	1.70 (2)	2.5763 (12)	163 (2)
O10—H10*O*···O2^ii^	0.91 (2)	1.72 (2)	2.6077 (12)	163 (2)
O12—H12*O*···O3^iii^	0.90 (2)	1.71 (2)	2.5978 (12)	170.9 (19)
C16—H16···O4	0.93	2.53	3.3333 (15)	144
C44—H44···O4^iv^	0.93	2.43	3.2404 (17)	145
C52—H52···O11^v^	0.93	2.49	3.3231 (16)	149
C62—H62···O11^i^	0.93	2.51	3.367 (2)	153
C64—H64···O5^vi^	0.93	2.46	3.263 (2)	144
C68—H68···O3^vi^	0.93	2.55	3.2747 (17)	135
C71—H71···O5	0.93	2.59	3.2765 (18)	131
C75—H75···O2^i^	0.93	2.41	3.1184 (16)	133
C96—H96···O1	0.93	2.49	3.1832 (15)	132

Symmetry codes: (i) *x*+1, *y*, *z*; (ii) *x*+1, *y*, *z*+1; (iii) *x*, *y*, *z*+1; (iv) *x*−1, −*y*+1/2, *z*−3/2; (v) *x*, −*y*+1/2, *z*−3/2; (vi) *x*, −*y*+1/2, *z*−1/2.
